# Protective or limited? Maternal antibodies and RSV-associated lower respiratory tract infection in hospitalized infants aged 28-90 days

**DOI:** 10.3389/fimmu.2024.1437616

**Published:** 2025-01-07

**Authors:** Shuanglian Li, Chenghao Mei, Sainan Chen, Chenglin Wang, Yelei Gao, Jinhua Ma, Li Zhong, Tingting Luo, Xin Zhao, Huaqin Bu, Ying Lyu, Xiaohu Kuang, Zhenxing Jia, Xiaoli Wang, Yuqing Wang, Daiyin Tian

**Affiliations:** ^1^ Department of Respiratory Medicine, Children’s Hospital of Chongqing Medical University, National Clinical Research Center for Child Health and Disorders, Ministry of Education Key Laboratory of Child Development and Disorders, Chongqing, China; ^2^ Chongqing Key Laboratory of Child Rare Diseases in Infection and Immunity. Children's Hospital of Chongqing Medical University, Chongqing, China; ^3^ Department of Respiratory Medicine, Children's Hospital of Soochow University, Soochow, China; ^4^ Department of Clinical Center and Department of mAbs Discovery Center, Zhuhai Trinomab Pharmaceutical Co., Ltd, Zhuhai, China; ^5^ Department of Respiratory Medicine, Yibin Hospital Affiliated to Children’s Hospital of Chongqing Medical University, Yibin, China

**Keywords:** RSV, Pre-F, Post-F, neutralizing antibody, maternal antibody, infants, severity of illness

## Abstract

**Background:**

Respiratory syncytial virus (RSV) is a major cause of severe health problems in newborns and young children. The protective role and limitations of serum maternal RSV antibodies in infants under 3 months remain controversial.

**Methods:**

A two-center prospective study from 2020 to 2023 recruited infants (n=286) admitted to the respiratory departments of two children’s hospitals in southwestern and southeastern China during RSV epidemic. These infants were hospitalized with lower respiratory tract infections (LRTI). We evaluated the relationship between serum RSV Prefusion (Pre-F), postfusion (Post-F) IgG levels, subtype neutralizing antibodies, and the incidence of RSV infection, as well as the relationship between these maternal antibodies and severity of disease. Since this prospective study only included data from RSV epidemic, we retrospectively reviewed medical records from the Children’s Hospital of Chongqing Medical University for the years 2019 to 2023 (n=3467) to analyze population characteristics during both RSV epidemic and non-epidemic periods, using the same inclusion and exclusion criteria.

**Result:**

There were no significant differences in RSV Pre-F IgG, Post-F IgG, or RSV A or B neutralizing antibody levels between the RSV infected and non-infected groups during the epidemic. While RSV Pre-F IgG antibody was inversely correlated with disease severity, RSV Post-F IgG, and RSV A and B neutralizing antibodies did not show a similar correlation across the three illness severity categories. Additionally, there were no differences in age, gender, or illness severity distribution among hospitalized patients during epidemic and non-epidemic periods.

**Conclusion:**

Serum maternal antibody levels offer insufficient protection against RSV-associated LRTI in hospitalized infants aged 28 to 90 days.

## 1 Introduction

Respiratory syncytial virus (RSV) is a globally prevalent pathogen and the leading cause of lower respiratory tract infection (LRTI) in children under the age of 5 years (1). Severe RSV infection often occurs in children under 6 months of age (2), with age under 3 months being an independent predictor of RSV infection (3). Researching RSV in this vulnerable population is crucial for improving clinical outcomes and developing effective preventive strategies.

Due to the immaturity of the immune system, infants are at risk of antibody dependent enhancement (ADE) following RSV vaccination (4). Currently, passive immunoprophylaxis with monoclonal antibodies is the primary method of immunization against RSV in young children (5). However, the widespread use of palivizumab is limited by its high cost and other constraints, particularly in low- and middle-income countries (6). Recent research aims to immunize pregnant women with the RSV prefusion (Pre-F) protein vaccine to offer high levels of maternal antibodies to newborns (4, 7, 8).

As of August 2023, the only FDA-approved RSV Pre-F protein vaccine for pregnant women, PF-06928316 (Abrysvo), has been shown to reduce the incidence of RSV-associated LRTI and severe RSV-associated LRTI in infants up to 180 days old in its Phase III clinical trial (NCT04424316) (4). However, RSV Pre-F vaccination did not prevent medically attended RSV-associated LRTI within 90 days after birth. This suggest that infants under 90 days may represent a special population for RSV prevention.

The protective role of maternal antibodies against RSV infection in infants without vaccine or antibody intervention remains controversial, largely due to variations in population selection and testing methods used in previous studies (5, 9, 10). The levels of serum RSV neutralizing antibodies in non-intervention infants are significantly lower than those in infants who have received maternal RSV vaccination during pregnancy or RSV antibody after birth (11). In this study, we investigated the potential association between maternal antibodies and RSV-associated LRTI, as well as disease severity in infants aged 28- 90 days who had not received vaccine interventions. The results of this study aim to inform the development of more effective preventive strategies for this vulnerable population.

## 2 Materials and methods

### 2.1 Study design

A two-center prospective study was conducted from January 2020 to December 2023 during RSV epidemic period, recruiting children hospitalized in the respiratory wards of Children’s Hospital of Chongqing Medical University and Children’s Hospital of Soochow University, China. A total of 317 subjects were recruited, with 286 children ultimately included based on the inclusion and exclusion criteria. Serum samples were collected from all children on the day of admission, and they were categorized into RSV-infected and non-RSV-infected groups based on RSV antigen or nucleic acid test results. Medical records, including baseline information (sex, age, admission time, number of siblings, feeding pattern, RSV antigen or nucleic acid results, and other clinical signs and laboratory tests for disease severity), were also collected.

Inclusion criteria:(1) Children under 3 months of age hospitalized with a diagnosis of pneumonia or bronchiolitis; (2) No previous history of RSV infection; (3) Have had RSV antigen or nucleic acid testing; Exclusion criteria: (1) Children with severe deformities; (2) Bronchopulmonary dysplasia; (3) Primary immunodeficiency diseases; (4) Gestational age under 37 weeks.

In addition, a retrospective study was conducted from January 2019 to July 2023 at the Children’s Hospital of Chongqing Medical University. We included 3467 patients who also met the above inclusion and exclusion criteria, using the hospital’s medical record system to collect the same medical records items.

### 2.2 Serum RSV Pre-F and Post-F IgG antibodies detection

A modified indirect ELISA assay was used to measure IgG antibody titers to RSV Pre-F and postfusion (Post-F) proteins as previously described (12). In brief, High-affinity 96-well plates (Thermo #442414) were coated with 2 µg/mL of RSV Pre-F or Post-F proteins at 4°C for 12h. The plates were then washed with phosphate-buffered saline (PBS) containing 0.05% Tween 20, followed by blocking with a buffer composed of 1.5% goat serum, 4g/ml Whey Protein, 0.005% Tween 20, and 5% 20x PBS at 37°C for 2 hours. Serum samples, diluted in blocking buffer, were added to the plates, which were then washed and incubated with the secondary antibody (Abcam #205719) for 1 hour. After additional washes with phosphate-buffered saline, TMB single-component substrate solution (Solarbio #PR1200) was added for color development. The reaction was terminated with sulfuric acid, and absorbance was quantified at 450 nm and 630 nm by Enzyme meter. A blank control (without serum) was included, and the end titration was defined as the reciprocal of the previous serum dilution of the last serum dilution with an optical density (OD) value similar to the blank control.

### 2.3 Micro-neutralization test

To standardize results and minimize discrepancies due to variations in neutralization assays across different institutions (13, 14). we established the following protocol to detect RSV A and RSV B neutralizing antibodies. Serum samples were first heat-inactivated at 56°C for 30 minutes, then diluted two-fold in 96-well plates, with dilutions ranging from 1:5 to 1:640 in a final volume of 50 µL. Equal amounts of RSV A2 or RSV B9320 were added to the wells, and the mixtures were incubated at 37°C with 5% CO2 for 1 to 2 hours. Following incubation, 100 µL of 2% FBS medium containing 1x10^5/mL HEp-2 cells was added to each well, and the plates were incubated at 37°C with 5% CO2 for 3 to 5 days. Control wells included cell-only controls and virus-only wells (virus + cells). After incubation, the wells were washed with 0.1% PBST, and cells were fixed with 80% acetone (100 µL/well) for 15 minutes at room temperature. After another wash, the plates were blocked with blocking solution for 2 hours at 37°C. A primary antibody against RSV fusion protein (Abcam #24011) and the corresponding secondary antibody (Abcam #205719) were added and incubated for 1 hour. Following washes, color development reagent and termination solution were applied, and absorbance was measured at 450 nm and 630 nm using an ELISA meter. For the RSV A neutralization assay and RSV B titer conversion, we used the World Health Organization’s (WHO) first international standard anti-RSV serum (NIBSC# 16/284). The results are expressed as log_2_ IU ml^-1^.

### 2.4 RSV illness severity

Medical records were collected to assess the severity of RSV disease using three different scoring system. (1) Score 1: The Bronchiolitis severity score (**Supplementary Table S1**) (15), a widely used tool in Chinese pediatric clinical practice; (2) Score 2: Wang’s Score (**Supplementary Table S2**) (16), an internationally recognized scoring system for RSV severity; (3) Score 3: The Acute bronchiolitis severity scale (**Supplementary Table S3**) (17), which takes into account respiratory and heart rates in young infants and excludes oximetry as an indicator for advanced RSV symptoms. This scale is particularly suited for evaluating LRTI in younger infants in our study. Details of these scoring systems are shown in the **Supplementary Material**.

### 2.5 Definition of periods of high RSV detection rate

Seasonal variations in RSV positivity among the enrolled children were analyzed using the Average Annual Percentage (AAP) method (18) to identify periods of high detection rates. In brief, the monthly number of RSV-positive cases was ranked from highest to lowest, and the months accounting for the first 75% of the total RSV-positive cases in a given epidemic year were defined as the period of high detection rate. The formula used was:


$$AAP_{i}=\frac{{\text{n}}_{\text{i}}}{\sum_{1}^{12}{\text{n}}_{\text{i}}}\times 100\%$$

Where i represents the month and n represents the number of RSV cases. Each RSV prevalence year was defined as the period from August 1 to July 31 of the following year.

### 2.6 Data information analysis

Baseline information and sample test data were primarily managed and analyzed using Excel. Component ratios were tested using the chi-square test or fisher’s exact test. Age distribution was calculated using Excel for proportions, and SPSS software was employed for two independent samples t-test. Paired-sample t-tests were performed to examine differences between the RSV-infected and non-RSV-infected groups, following a normality check for RSV Pre-F, Post-F IgG antibody and RSV A and B neutralizing antibody levels. R 4.2.0 was employed to plot frequency curves of antibody levels in both RSV-infected and non-RSV-infected groups. The relationship between antibody levels and age, RSV prevalence trends and RSV subtype neutralizing antibody levels, and changes in antibody titers and disease severity scores were analyzed using linear regression in GraphPad Prism and tested with the Spearman method. Statistically significant was set at P < 0.05.

## 3 Results

### 3.1 Population characteristics

The two-center study included a total of 286 children ages 28 to 90 days hospitalized for LRTI (**Table 1**). Of these, 252 were from Children’s Hospital of Chongqing Medical University and 34 from Children’s Hospital of Soochow University. The RSV detection rate in this population was 45.5%. The mean age of the infants in both the RSV-infected and non-RSV-infected groups were 59 days. There were no significant differences between the two groups in terms of age, gender, presence of siblings, or feeding patterns.

**Table 1:** Baseline information on the population recruited for the two-center study.

	Total number (n=286)	Non-RSV infection group (n=156) (54.5%)	RSV infection group (n=130) (45.5%)	OR	95% CI	P#
Chongqing	252 (88.1%)	134 (85.9%)	118 (90.8%)	0.62	0.29-1.31	0.21
Soochou	34 (11.9%)	22 (14.1%)	12 (9.2%)			
Gender						
Male	190 (66.4%)	105 (67.3%)	85 (65.3%)	1.09	0.67-1.78	0.73
Female	96 (33.6%)	51 (32.7%)	45 (24.6%)			
Age						
28-30	10 (3.5%)	4 (2.6%)	6 (4.6%)	0.54	0.15-1.97	0.52*
31-60	147 (51.4%)	76 (48.7%)	71 (53.8%)	0.79	0.50-1.26	0.32
61-90	129 (45.1%)	76 (48.7%)	53 (40.8%)	1.38	0.86-2.21	0.18
Average age		59 ± 18	59 ± 18			
Siblings (≥1)	129 (45.1%)	65 (41.7%)	64 (49.2%)	1.36	0.85-2.17	0.20
Siblings (=0)	157 (45.9%)	91 (58.3%)	66 (50.8%)			
Feeding patterns						
Breast milk	123 (43.0%)	61(39.1%)	62 (47.7%)	–	–	0.26
Milk power	27 (9.4%)	14 (9.0%)	13 (10.0%)			
Mixed feeding	136 (47.5%)	81 (51.9%)	55 (42.3%)			

# Chi-square test, *Fisher test.

### 3.2 Serum RSV antibodies decay in infants and the positive correlation between Pre-F IgG and neutralizing antibodies

**Figure 1:**
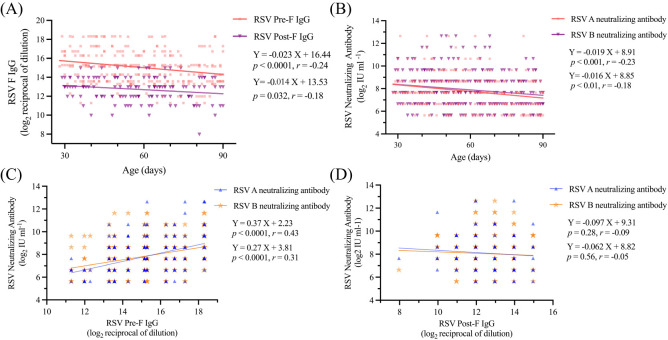
Serum antibodies of children decreased with the increase of age **(A)** Serum RSV Pre-F IgG in relation to age; **(B)** Serum RSV Post-F IgG in relation to age; **(C)** Serum RSV A and B neutralizing antibodies in relation to age; **(D)** Relationship between serum RSV F IgG antibody and neutralizing antibodies.

To analyze the changes in antibody levels among the 286 children, we first analyzed their serum antibody levels. The mean levels of RSV Pre-F IgG and Post-F IgG antibodies were 15 ± 1.7 and 12.7 ± 1.3 (log_2_ reciprocal of dilution), respectively. Additionally, the mean serum level of RSV A and RSV B neutralizing antibodies were 7.7 ± 1.5 and 7.9 ± 1.6 (log_2_ IU ml^-1^), respectively. By fitting the serum levels of RSV Pre-F and Post-F IgG antibodies, as well as RSV A and B neutralizing antibody levels to the ages of the 286 children, we observed that all four antibody titers declined with age (**Figures 1A, B**). The estimated half-lives for RSV Pre-F IgG, Post-F IgG, RSV A neutralizing, and RSV B neutralizing antibodies were 43, 71, 53 and 59 days, respectively, based on curve fitting to -1/a. Furthermore, we found a statistically significant positive correlation between serum RSV Pre-F IgG antibody and both RSV A and B neutralizing antibodies (P<0.0001, **Figure 1C**). However, no correlation was observed between RSV Post-F IgG antibody and neutralizing antibodies (**Figure 1D**), which is consistent with previous studies (19).

### 3.3 No difference in serum antibody levels between RSV-infected and non-infected children

**Figure 2:**
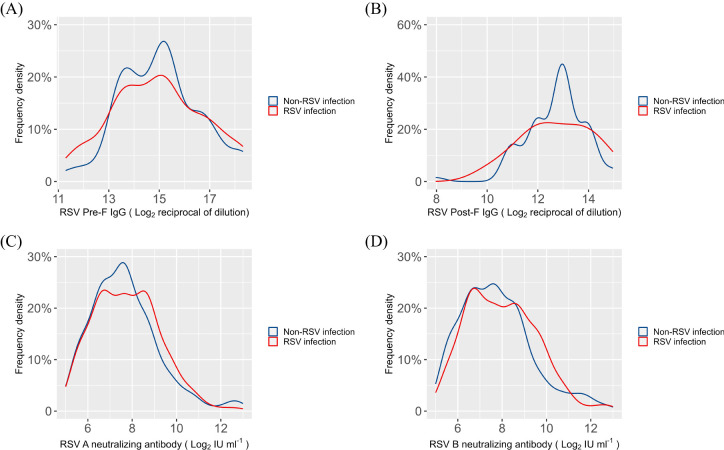
Density distribution curves of serum antibodies in RSV infection and non-RSV infection groups **(A)** RSV Pre-F IgG antibody; **(B)** RSV Post-F IgG antibody; **(C)** RSV A neutralizing antibody; **(D)** RSV B neutralizing antibody.

To evaluate the variations in serum antibody titers between the RSV-infected and non-RSV-infected groups, we analyzed the density distribution curves of RSV Pre-F and Post-F IgG antibodies, as well as RSV A and B neutralizing antibodies, in the 286 children (**Figure 2**). The results indicated no statistically significant differences in the levels of RSV Pre-F or Post-F IgG, nor in the levels of RSV A or B neutralizing antibody, between the two groups (P=0.59, 0.24, 0.84, 0.68, **Supplementary Table S4**).

### 3.4 Severity of RSV-associated LRTI negatively correlates with serum RSV Pre-F IgG antibody, but not with neutralizing antibodies.

**Figure 3:**
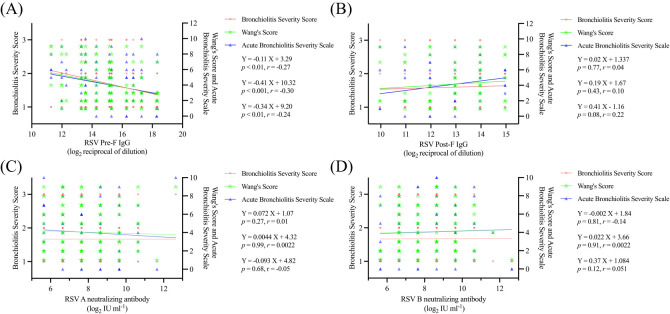
Relationship between serum antibodies and disease severity in children **(A)** Linear fit of serum RSV Pre-F antibodies to the three disease severity scoring modalities; **(B)** Linear fit of serum RSV Post-F antibodies to the three disease severity scoring modalities; **(C)** Linear fit of serum RSV A neutralizing antibodies to the three disease severity scoring modalities; **(D)** Linear fit of serum RSV B neutralizing antibodies to the three disease severity scoring modalities.

To further explore the relationship between serum RSV antibody levels and disease severity in young infants, we evaluated the severity of LRTI in 130 children from the RSV infection group using the three previously described scoring methods. Our analysis revealed a strong inverse correlation between RSV Pre-F IgG antibody levels and disease severity (all P values < 0.01), whereas no such association was observed for RSV Post-F IgG or RSV A/B neutralizing antibody (**Figure 3**). Additionally, based on a multicenter epidemiological study conducted in China during the same period (20), we identified the peak epidemic months for RSV subtypes A and B (**Supplementary Table S5**). During these RSV subtype epidemic months, no statistically significant association was found between disease severity and RSV A/B neutralizing antibody levels (**Supplementary Table S5**).

### 3.5 No difference in RSV-endemic and non-endemic periods in terms of RSV-infected populations and severity of illness

We analyzed monthly hospitalizations and RSV detection rates over nearly five years in the study population. A retrospective analysis of 3,467 children aged 27 to 90 days revealed an overall RSV detection rate of 27.5% (**Supplementary Figure S1**). Children hospitalized for RSV infection were more likely to have one or more siblings compared to controls (P = 0.017, **Supplementary Table S6**). No significant differences were found in age distribution or feeding patterns between the RSV-infected and non-RSV-infected groups (**Supplementary Figure S6**). Using the AAP method, we identified high RSV detection periods as follows: November 2019 to January 2020 (peak in December), November 2020 to March 2021 (peak in December), October 2021 to February 2022 (peak in January), and March 2023 to June 2023 (peak in April) (**Supplementary Figure S1**, **Supplementary Table S7**). The delayed RSV detection peak in the 2022–2023 data aligns with previous reports (21), potentially due to the use of non-pharmacological interventions (NPIs) in China between October and December 2022. Additionally, we assessed 798 RSV-infected children during this period to evaluated whether the population distribution of RSV infection was related to the epidemic period. Our findings indicated no significant changes in the distribution of age, sex, or disease severity between the epidemic and non-epidemic seasons (**Table 2**).

**Table 2:** Distribution of children by sex, age, and disease severity during epidemic and non-epidemic periods.

	Epidemic season (N=649)	Non-epidemic season (N=149)	OR	95% CI	P#
Male	404 (62.2%)	99 (66.4%)	1.20	0.83-1.75	0.34
Female	245 (37.8%)	50 (33.6%)			
Age					
27-30	16 (2.4%)	3 (2.0%)	–	–	0.12
31-60	367 (56.9%)	71 (47.7%)			
61-90	266 (41.0%)	75 (50.3%)			
Average age	60 ± 18	60 ± 18			
Siblings (≥1)	312 (48.1%)	67 (45.0%)	0.88	0.62-1.26	0.50
Siblings (=0)	337 (51.9%)	82 (55.0%)			
Feeding patterns					
Breast milk	289 (44.5%)	60 (40.3%)	–	–	0.62
Milk power	57 (8.8%)	15 (10.1%)			
Mixed feeding	303 (46.7%)	74 (49.7%)			
Score					
Score 1: Mild	241 (37.1%)	51 (34.2%)	1.14	0.78-1.65	0.51
Score 1: Moderate+	408 (62.9%)	98 (65.8%)			
Score 2: Mild	349 (52.8%)	86 (57.8%)	0.85	0.60-1.22	0.38
Score 2: Moderate+	300 (46.2%)	63 (42.3%)			
Score 3: Mild	352 (54.2%)	74 (49.7%)	1.20	0.84-1.72	0.31
Score 3: Moderate+	297 (45.8%)	75 (50.3%)			

Raw data

https://www.jianguoyun.com/p/DVd3xw8QtvHzDBiRsdYFIAA

# Chi-square test; Moderate+: Moderate and severe; Score 1: Bronchiolitis severity score; Score 2: Wang’s clinical score system; Score 3: Acute bronchiolitis severity scale.

## 4 Discussion

Maternal antibodies are primarily transmitted to infants through the placenta and decline rapidly within two to three months after birth, typically disappearing by six months (22–25). Since newborns do not start producing IgG until 15 weeks of age (26), serum antibodies in infants under three months old largely reflect maternal antibody levels. In this prospective study, serum samples were collected from children aged 28 to 90 days upon their first hospital admission. Subsequently, the levels of four maternal antibodies were tested and the results were reasonably consistent: the ratio of RSV Pre-F and Post-F IgG antibodies was consistent with previous studies on children younger than two months (27); The half-lives of RSV A and B neutralizing antibodies, as fitted to age, were 54 and 60 days, respectively, which is in line with other studies reporting half-lives ranging from 21 to 79 days (9, 28–30).

RSV Pre-F vaccination during pregnancy is not effective in preventing RSV-LRTI in children younger than 3 months of age, although it does provide protection for children up to 6 months of age (4). Most research on unvaccinated children has focused on infants six months or older (5, 29, 31, 32), with only a few studies exploring the relationship between serum antibodies and RSV disease in infants younger than 90 days, resulting in inconsistent findings (9, 27). For example, Buchwald et al. studied 587 infants during their first three months and found that RSV serum neutralizing antibody levels in non-hospitalized healthy children were higher than those in children with RSV infection or infants with influenza symptoms without RSV infection. However, no difference was noted between the latter two groups, and RSV fusion antibody levels were not reported (9). In contrast, Capella et al. conducted a prospective study in children under three months and reported no difference in serum neutralizing antibody levels between RSV-infected and healthy control groups, though the sample size was small (n=22) (27). Similarly, our study found no statistically significant difference in serum antibody levels between the RSV-infected and non-RSV-infected groups in children aged 28 to 90 days.

Previous studies have shown inconsistent findings regarding the impact of RSV maternal antibody levels on disease severity. Some studies suggest a protective effect: Except for animal study (33), in a Qatari serologic study of 65 mother-infant pairs, neutralizing antibody titers were significantly negatively correlated with the severity of infection in infants younger than six months, although RSV Pre-F IgG antibody was not associated with disease severity based on Wang’s Score (31). Inversely, a study of 139 U.S. children under ten months infected with RSV found that RSV fusion antibody was significantly negatively correlated with disease severity, while RSV A and B neutralizing antibodies showed no associated using the Global respiratory severity score (GRSS) (32).

Maternal serum during pregnancy and cord blood have been used to study the relationship between maternal antibodies and disease severity of RSV infection, although the results remain controversial. Several studies suggested that RSV-specific antibody levels in maternal and cord blood during pregnancy provide a protective effect for young infant. For instance, lower serum maternal Pre-F IgG antibody in early pregnancy (9-12 weeks) have been associated with an increased risk of severe RSV infection in offspring up to three months of age (12). However, maternal immunity provides only temporary protection to neonates through the transplacental transfer of maternal IgG antibodies, which begins around 13 weeks of gestation and increases throughout pregnancy, with the majority of transfer occurring in the last trimester (34). This raises questions about whether early trimester maternal blood can accurately represent maternal antibody levels. Several studies of cord blood have found a negative association between neutralizing antibody level and RSV infection (n=90, 1561, 169) (5, 35, 36) as well as disease severity (n=643) (37). In contrast, other studies found no association between cord blood neutralizing antibody levels and severe RSV infection (n=169, 310) (5, 10). Importantly, these studies involved children up to 6 months of age. A prospective study focusing on infants younger than three months (n=587) (9) revealed that higher RSV A neutralizing antibody level in cord blood were significantly associated with a reduced incidence of pneumonia in infants, whereas no difference was observed in the potency of RSV B neutralizing antibodies between case and control group. This suggests that the protective properties of cord blood antibodies may not be generalized across all RSV subtypes.

The discrepancies in the relationship between maternal antibodies and disease severity could be attributed to differences in the age range, sample size of the populations studied, and variations in testing methods. To minimize bias, our study used three scoring methods to assess the relationship between maternal antibodies and disease severity in hospitalized children with LRTI. Additionally, we introduced an innovative approach by utilizing standard neutralizing antibody serum to standardize the reporting of neutralizing antibody level (14). These methods not only allow for direct comparison of results across studies but also provide a valuable reference for future research. Furthermore, only one other serological investigation on infants younger than three months has utilized a similar methodology (38), and our findings align with those from the placebo group in that study.

Our study demonstrated that only serum RSV Pre-F IgG antibody levels exhibited a statistically significant inverse correlation with the severity of RSV-LRTIs, while RSV Post-F IgG antibody or RSV A/B neutralizing antibody was not associated with disease severity. This inconsistency between maternal antibodies and disease severity warrants further reflection. Neutralizing antibody titers are commonly used to describe the ability of an antibody to neutralize virus. Previous studies (19, 39) have reported that the quantity of site RSV Ø–specific antibody correlated with neutralizing activity, whereas the magnitude of binding antibody competing at site II does not correlate with neutralization. Cristina Capella SC, et al. found that RSV Pre-F protein adsorption eliminated neutralizing activity in serum samples from all patients (55%-100%), whereas a higher proportion of neutralizing activity was lost with RSV G protein adsorption (0%-45%) compared to RSV post-F (0%-29%). Moreover, higher concentrations of Pre-F and G antibodies, rather than Post-F antibodies, were associated with lower clinical disease severity scores (27). Therefore, it remains to be investigated whether other factors, such as antibodies against the RSV G protein, contribute to the different trends observed between Pre-F IgG and neutralizing antibody in relation to disease severity. Furthermore, another potential explanation for the lack of correlation between neutralizing antibody levels and disease severity could be that the neutralizing antibody titers in unvaccinated infants younger than three months may not yet reach the protective threshold. This concept has been previously suggested by other researchers (9, 40). Notablly, cord blood from pregnant women in the unvaccinated group exhibited significantly lower RSV neutralizing antibody level compared to that of the vaccinated group, being approximately 9% of the latter (11).

As our prospective study only included children enrolled during the 2020 to 2023 RSV epidemic, we extended our investigation through a retrospective analysis covering the entire period from January 2019 to July 2023. This analysis revealed no significant differences in the population characteristics affected by RSV or in disease severity between epidemic and non-epidemic periods. Notably, the characteristics of populations during non-endemic periods of RSV have not been previously reported. The retrospective study suggested that the conclusions drawn from prospective studies conducted exclusively during epidemics may be generalizable to year-round populations. However, given that the relationship between viral load and disease severity remains inconsistent across studies (41, 42), it would be premature to conclude that there is no variation in RSV virulence between epidemic and non-epidemic periods. Further research is necessary to explore this issue in greater detail.

Our study has several limitations. First, we did not include children who were not hospitalized, and the inclusion criteria for hospitalized children were based solely on the absence of a prior RSV diagnosis. This means that children who may have had mild, undiagnosed RSV infections in the past were not accounted for. Additionally, we did not collect data on gestational age, which previous studies have identified as a key determinant of RSV disease severity (43). Furthermore, we did not differentiate between RSV subtypes in the infants, which is important given that previous study involving children aged 0-5 months have shown variations in neutralizing antibody levels following infections with different RSV subtypes (13). Although our study incorporates data on prevalent subtypes in China, regional bias may still exist. Moreover, we did not analyze other viral infections in the study population, despite previous research indicating that approximately 29.2% of children with RSV-LRTI also have co-infections with other viruses (44). However, no significant correlation was found between the severity of illness and co-infection in RSV-infected children with bronchiolitis (45), suggesting that co-infections may not interfere with the results of our study. Lastly, our study recruited only children from the respiratory unit, excluding those aged 0-27 days.

In conclusion, this two-center prospective study underscores the insufficient protection provided by maternal antibodies in infants aged 28 to 90 days, while also offering insights into the clinical epidemiology of RSV in this age group. Future RSV prevention strategies should consider the unique immunity needs of 3-month-old infants.

## Data Availability

The original contributions presented in the study are included in the article/**Supplementary Material**. Further inquiries can be directed to the corresponding authors.
