# Supplementary material for: Protective or limited? Maternal antibodies and RSV-associated lower respiratory tract infection in hospitalized infants aged 28-90 days
Source: Front Immunol. 2025 Jan 7;15:1437616. doi: 10.3389/fimmu.2024.1437616 (PMC11753235; doi:10.3389/fimmu.2024.1437616)
Supplement: Supplementary file 1 [file DataSheet1.pdf]

Supplementary Material

Protective or Limited? Maternal Antibodies and RSV-associated Lower Respiratory Tract Infection in Hospitalized Infants Aged 28-90 days

Shuanglian Li[1,2†], Chenghao Mei[1,2†], Sainan Chen[3], Chenglin Wang[1,2,] Yelei Gao[1,2], Jinhua Ma[1,2], Li Zhong[1,2], Tingting Luo[1,2], Xin Zhao[1,2], Huaqin Bu[1,2], Ying Lyu[1,2], Xiaohu Kuang[4], Zhenxing Jia[4], Xiaoli Wang[4], Yuqing Wang[3* †], Daiyin Tian[1,2,5* †]

*** Correspondence:** Yuqing Wang[2†] and Daiyin Tian[1,3†]:wang_yu_qing@126.com and t_dy@163.com. These authors contributed equally to this work and share first correspondence authorship.

1 Supplementary Figures and Tables

1.1 Supplementary Figures

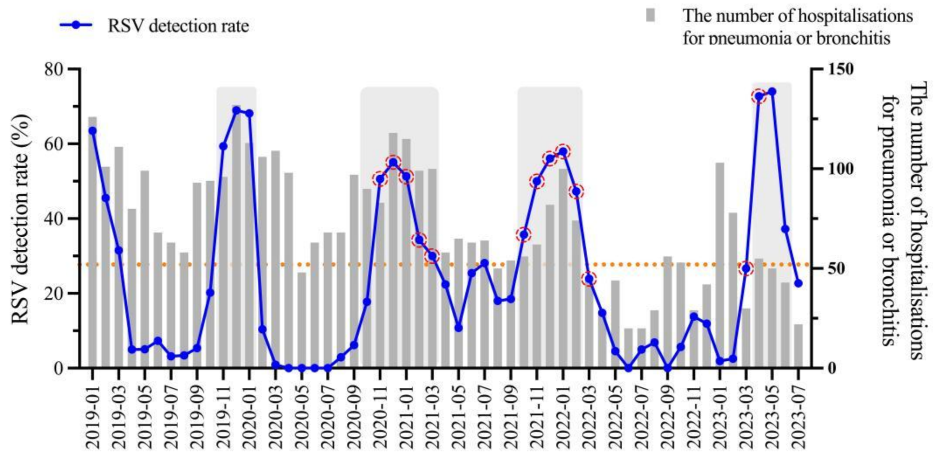

Supplementary Figure 1. RSV detection and total hospitalizations among 3467 hospitalized children aged 27-90 days, by month of hospitalization, 2019-2023.

The orange line represents the RSV detection rate of 27.5% in this retrospective study, and the red circle represents the month of hospitalization corresponding to the 286 children recruited prospectively. Gray shading corresponding to the month is the month of prevalence according to the AAP method.

1.2 Supplementary Table

Supplementary Table 1. Bronchiolitis severity score

Item	Mile	Moderate	Severe
Feeding volume	Normal	Down to less than half of normal	Down to more than half of normal or food refusal
Respiratory frequency	Normal or slightly faster	>60 times/min	>70 times/min
Three depression sign	Mild or no sign	Moderate (abvious)	Severe (strikingly apparent)
Nasal flapping or moaning	None	None	Yes
Oxygen saturation of blood	>92%	88～92%	<88%
Mental state	Normal	Mild or intermittent irritability	Extreme restlessness, drowsiness, coma

Note: Moderate to severe bronchiolitis can be determined if any of the criteria exist

Supplementary Table 2. Wang's clinical score system

Items	Points			
	0	1	2	3
Respiratory frequency（times/min）	<30	30-45	46-60	>60
Wheezing	None	Terminal expiration or only with stethoscope	Entire expiration or audible on expiration without stethoscope	Inspiration and expiration without stethoscope
Retraction	None	Intercostal recession	Trachea-sternal recession	Severe with nasal flow
General condition	Normal	-	-	Irritable/lethargic/poor feeding

Note: Mild, 0~4 points; Moderate, 5 to 8 points; Severe, 9 to 13 points

Supplementary Table 3. Acute bronchiolitis severity scale

Items*	Points				
	0	1	2	3	4
Wheezing	No	Wheezing at end of expiration	Wheezing throughout expiration	Ins-expiratory wheezing	Hypoventilation
Crackles	No	Crackles in one field	Crackles in 2 fields	Crackles in 3 fields	Crackles in 4 fields
Effort	No	Subcostal or lower intracostal retractions	+suprasternal retractions or nasal flaring	+nasal flaring and suprasternal (universal)	-
I：E ratio	Normal	Symmetrical	Inverted		
Respiratory rate					
<2 months	<57	57-66	>66		
2-6 months	<53	53-62	>62		
Heart rate					
7day-2 months	125-152	153-180	>181		
2-12 months	120-140	141-160	>161		

Note: Mild, 0 ~ 4 points; Moderate, 5 ~ 9 points; Severe, 10 ~ 13 points

*Interpretation of the exploration on the Acute bronchiolitis severity scale

Wheezing: We assess the presence of wheezing at the end of or throughout expiration and whether it is also audible during inspitation. In cases of severe obstruction hypoventilation without wheezing is assessed;

Crackles: We assess the extent of clearly audible crackles in the chest in all respiratory cycles in each lung in both the anterior and posterior fields;

Efforts: We cumulatively assess the level of established and persistent respiratory effort from subcostal retractions to universal retractions, as shown in the table;

I:E ration: We assess whether the ration of inspiration with respect to expiration is normal, symmetrical or inverted with the lengthening of expiration time;

RR: rate over the course of a minute without interference from coughing, crying or apnoeas, according to age;

HR: Heart rate for one minute in baseline situation, according to age.

Supplementary Table 4. Comparison of serum antibody levels between RSV infection group and non-RSV infection group

	Non-RSV infection group， N	RSV infection group， N	*P#*
RSV A neutralizing antibody	7.73 (1.55)，N=156	7.83 (1.45)，N=129	0.59
RSV B neutralizing antibody	7.64 (6.64, 8.64)，N=155	7.64 (6.64, 8.64)，N=129	0.24
RSV Pre-F IgG	15.07 (1.63)， N=151	15.02 (1.83)， N=124	0.84
RSV Post-F IgG	12.71 (1.18), N=71	12.72 (1.40), N=64	0.99

#Independent-samples T test

Data are normally distributed and expressed as mean (standard deviation) or Median (IQR); N: number; Units: RSV A、B neutralizing (log_2 IU ml^{-1}), RSV Pre-F and Post-F IgG (log_2 reciprocal of dilution). Due to the limited amount of serum collected from children, only 285 children were tested for RSV A neutralizing antibody levels, 284 children were tested for RSV B neutralizing antibody levels, 275 children were tested for serum RSV F IgG antibody levels, and 135 children were tested for serum RSV F IgG antibody levels.

Supplementary Table 5. According to the epidemic subtypes in article, we only listed the RSV epidemic subtypes corresponding to the months of our prospective study, and the correlation analysis between the level of neutralizing antibody corresponding to RSV subtype and the severity of disease。

Months	Nov. 2020	Dec. 2020	Jan. 2021	Feb. 2021	Mar. 2021	Oct. 2021	Nov. 2021	Dec. 2021	Jan. 2022	Feb. 2022	Mar. 2022	Dec. 2022	Mar. 2023	Apr. 2023
RSV subtype	A	B	B	B	B	B	A	A	A	B	B	NA	A	NA

	Levels of RSV A neutralizing antibodies corresponding to months of RSV A prevalence		Levels of RSV B neutralizing antibodies corresponding to months of RSV B prevalence	
	|r|	P	|r|	P
Score 1	0.10	0.44	0.08	0.55
Score 2	0.06	0.65	0.04	0.77
Score 3	0.09	0.51	0.13	0.29

Pearson correlation analysis was used, and the figure was the absolute value of correlation coefficient (r) and P value. Score 1: Bronchiolitis Severity Score, Score 2: Wang's Score, Score 3: Acute Bronchiolitis Severity Scale.

Supplementary Table 6. Baseline information on the population recruited for the retrospective study

	Total number (N=3467)	Non-RSV infection group (n=2511) (72.4%)	RSV infection group (n=956) (27.6%)	OR	95% CI	P#
Gender						
Male	2125 (61.3%)	1528 (60.9%)	597 (62.4%)	1.070	0.918-1.247	0.389
Female	1342 (38.7%)	983 (39.1%)	359 (37.6%)			
Age (days)						
27-30	103 (3.0%)	82 (3.3%)	21 (2.2%)	1.503	0.925-2.442	0.098
31-60	1928 (55.6%)	1413 (56.3%)	515 (53.9%)	1.102	0.949-1.280	0.203
61-90	1436 (41.4%)	1016 (40.5%)	420 (43.9%)	0.867	0.746-1.008	0.064
Average age		60 ± 19	60 ± 18			
Siblings (⩾1)	1555 (44.9%)	1095 (43.6%)	460 (48.1%)	1.199	1.033-1.393	0.017
Siblings (=0)	1912 (55.1%)	1416 (56.4%)	496 (51.9%)			
Feeding patterns						
Breast milk	1529 (44.1%)	1109 (44.2%)	420 (43.9%)	-	-	0.914

Milk power	329 (9.5%)	241 (9.6%)	88 (9.2%)
Mixed feeding	1609 (46.4%)	1161 (46.2%)	448 (46.9%)

Chi-square test

Supplementary Table 7. The monthly number of patients and RSV infections in the retrospective study from Aug. 2019 to Jul. 2023. According to the AAP method, the RSV epidemic months were highlighted.

month/year	Aug. 2019 - Jul. 2020			Aug. 2020- Jul. 2021			Aug. 2021- Jul. 2022			Aug. 2022- Jul. 2023		
	Number of patients	Number of RSV infection	AAPi	Number of patients	Number of RSV infection	AAPi	Number of patients	Number of RSV infection	AAPi	Number of patients	Number of RSV infection	AAPi
8	53	2	0.88%	60	2	0.74%	46	9	4.62%	22	2	1.79%
9	80	5	2.21%	83	4	1.48%	48	8	4.10%	50	0	0.00%
10	84	16	7.08%	72	11	4.07%	45	17	8.72%	44	2	1.79%
11	83	48	21.24%	77	38	14.07%	48	25	12.82%	24	4	3.57%
12	118	80	35.40%	102	57	21.11%	65	39	20.00%	32	4	3.57%
1	91	63	27.88%	103	51	18.89%	78	49	25.13%	91	2	1.79%
2	96	11	4.87%	88	33	12.22%	69	32	16.41%	73	2	1.79%

3	106	1	0.44%	92	26	9.63%	37	9	4.62%	26	8	7.14%
4	87	0	0.00%	50	11	4.07%	25	4	2.05%	51	38	33.93%
5	45	0	0.00%	53	6	2.22%	38	2	1.03%	42	31	27.68%
6	52	0	0.00%	56	15	5.56%	18	0	0.00%	40	14	12.50%
7	60	0	0.00%	57	16	5.93%	16	1	0.51%	21	5	4.46%
Total number		226			270			195			112	

Supplementary Table 8. Monthly number of patients and RSV infection and detection rate from Jan. 2019 to Jul. 2023 in the retrospective study

month/year	Jun. 2019 - Jul. 2019			Aug. 2019 - Jul. 2020			Aug. 2020- Jul. 2021			Aug. 2021- Jul. 2022			Aug. 2022- Jul. 2023		
	Number of patients	Number of RSV infection	RSV detection rate	Number of patients	Number of RSV infection	RSV detection rate	Number of patients	Number of RSV infection	RSV detection rate	Number of patients	Number of RSV infection	RSV detection rate	Number of patients	Number of RSV infection	RSV detection rate
8	—	—	—	53	2	3.77%	60	2	3.33%	46	9	19.57%	22	2	9.09%
9	—	—	—	80	5	6.25%	83	4	4.82%	48	8	16.67%	50	0	0.00%
10	—	—	—	84	16	19.05%	72	11	15.28%	45	17	37.78%	44	2	4.55%

11	—	—	—	83	48	57.83%	77	38	49.35%	48	25	52.08%	24	4	16.67%
12	—	—	—	118	80	67.80%	102	57	55.88%	65	39	60.00%	32	4	12.50%
1	110	70	63.64%	91	63	69.23%	103	51	49.51%	78	49	62.82%	91	2	2.20%
2	88	40	45.45%	96	11	11.46%	88	33	37.50%	69	32	46.38%	73	2	2.74%
3	89	29	32.58%	106	1	0.94%	92	26	28.26%	37	9	24.32%	26	8	30.77%
4	72	4	5.56%	87	0	0.00%	50	11	22.00%	25	4	16.00%	51	38	74.51%
5	88	4	4.55%	45	0	0.00%	53	6	11.32%	38	2	5.26%	42	31	73.81%
6	67	5	7.46%	52	0	0.00%	56	15	26.79%	18	0	0.00%	40	14	35.00%
7	56	1	1.79%	60	0	0.00%	57	16	28.07%	16	1	6.25%	21	5	23.81%
Total number	570			955			893			533			516		
